# Sequential Push-Pull Pumping Mechanism for Washing and Evacuation of an Immunoassay Reaction Chamber on a Microfluidic CD Platform

**DOI:** 10.1371/journal.pone.0121836

**Published:** 2015-04-08

**Authors:** Tzer Hwai Gilbert Thio, Fatimah Ibrahim, Wisam Al-Faqheri, Norhayati Soin, Maria Kahar Bador, Marc Madou

**Affiliations:** 1 Department of Biomedical Engineering, Faculty of Engineering, University of Malaya, 50603 Kuala Lumpur, Malaysia; 2 Centre for Innovation in Medical Engineering, Department of Biomedical Engineering, Faculty of Engineering, University of Malaya, 50603 Kuala Lumpur, Malaysia; 3 Faculty of Science, Technology, Engineering and Mathematics, INTI International University, Persiaran Perdana BBN, Putra Nilai, 71800 Nilai, Negeri Sembilan, Malaysia; 4 Department of Electrical Engineering, Faculty of Engineering, University of Malaya, 50603 Kuala Lumpur, Malaysia; 5 Department of Medical Microbiology, Faculty of Medicine, University of Malaya, 50603 Kuala Lumpur, Malaysia; 6 Department of Biomedical Engineering, University of California Irvine, Irvine, 92697, California, United States of America; 7 Department of Mechanical and Aerospace Engineering, University of California Irvine, Irvine, 92697, California, United States of America; University of Illinois at Chicago, UNITED STATES

## Abstract

A centrifugal compact disc (CD) microfluidic platform with reservoirs, micro-channels, and valves can be employed for implementing a complete immunoassay. Detection or biosensor chambers are either coated for immuno-interaction or a biosensor chip is inserted in them. On microfluidic CDs featuring such multi-step chemical/biological processes, the biosensor chamber must be repeatedly filled with fluids such as enzymes solutions, buffers, and washing solutions. After each filling step, the biosensor chamber needs to be evacuated by a passive siphoning process to prepare it for the next step in the assay. However, rotational speed dependency and limited space on a CD are two big obstacles to performing such repetitive filling and siphoning steps. In this work, a unique thermo-pneumatic (TP) Push-Pull pumping method is employed to provide a superior alternative biosensor chamber filling and evacuation technique. The proposed technique is demonstrated on two CD designs. The first design features a simple two-step microfluidic process to demonstrate the evacuation technique, while the second design shows the filling and evacuation technique with an example sequence for an actual immunoassay. In addition, the performance of the filling and evacuation technique as a washing step is also evaluated quantitatively and compared to the conventional manual bench top washing method. The two designs and the performance evaluation demonstrate that the technique is simple to implement, reliable, easy to control, and allows for repeated push-pulls and thus filling and emptying of the biosensor chamber. Furthermore, by addressing the issue of rotational speed dependency and limited space concerns in implementing repetitive filling and evacuation steps, this newly introduced technique increases the flexibility of the microfluidic CD platform to perform multi-step biological and chemical processes.

## Introduction

The centrifugal microfluidic compact disc (CD) platform is a platform that allows for miniaturization and automation of complex diagnostic processes [1–3]. At the core of the platform is a plastic disc that performs biological / chemical processes through the flow sequencing of small volumes of fluids through a network of interconnected micro chambers and channels embedded within the disc. As the CD is rotated, the generated centrifugal force causes passive pumping of liquid from the CD center towards the CD edge, while placement of passive valves at predetermined locations impedes liquid movement towards the CD edge. For liquid to move past, or burst through a passive valve, the centrifugal force must exceed the capillary force of the valve, which depends on the material, dimension, and relative location of the valve from the CD center [4]. Proper control of material, dimension, and relative location of the valve from the CD center and rotational speed then allows the liquid to burst through passive valves at predetermined burst frequencies (in revolutions per minute, rpm). This simple balancing of forces allows for simple CD designs without external physical connectors for fluid pumping and this increases the portability and disposability of the platform.

The flexibility in the design of the network of micro chambers, channels and valves allows for the implementation of a wide range of processes such as mixing, metering, siphoning, etc [1–3]. Some example complex processes include antigen and antibody detection assays [5–9], cell lysis and plasma separation [3,10], liver function screening [11] and polymerase chain reaction (PCR) [12,13].

On microfluidic CDs that have been developed for antigen and antibody detection, the biosensor chamber is either coated for immuno-interaction or a biosensor chip is embedded in it. The biosensor chamber is repeatedly filled and emptied with fluids such as test samples, buffers, reagents, and washing solutions, and as each specific fluid fills the biosensor chamber, it is either directly evacuated into a waste chamber through siphoning, or concurrently washed away with a washing solution while being siphoned [5–9]. However, siphoning depends on the rotational speed, requires a hydrophilic channel to operate, and is not easy to repeat as the channel property changes once the siphon has been primed and the channel wall is now wet. Furthermore, as the burst frequency of valves depends partly on the relative valve position from the CD center, this limits the number of valves which can be designed on the CD, and in turn limits the number of steps that can be carried out due to space constraints [14]. Because of these limitations most CDs proposed for complex assays perform limited washing steps only [5,7–9] or employ numerous active valves [6]. This inadvertently may result in improper washing of the biosensor chamber, resulting in poor specificity or sensitivity of the assays.

In this work, we describe simple to implement push-wash and pull-evacuation methods for filling and evacuating biosensor chambers on the CD platform using the thermo-pneumatic (TP) Push Pull pumping method developed by our group [14]. The method involves the thermo actuation of TP air chambers for push-wash (pushing liquid out from a connected source chamber) and pull-evacuation (pulling liquid out from an adjacent source chamber). The technique can be implemented regardless of the relative position of the source chamber from the CD, and has the following additional benefits: (i) simple to implement with just the addition of a forced convection heat source and temperature measurement apparatus, and with a heat source already available, heated incubation or polymerase chain reaction (PCR) can also be performed in situ on the CD platform, (ii) can be actuated on demand and easy to control, (iii) allows for numerous washing and evacuation cycles of the biosensor chamber and is limited only by the volume of the washing solution chamber, and (iv) no additional physical connectors that limits the portability of the CD platform are required.

To demonstrate the technique of push-wash and pull-evacuation, two CD designs were implemented. The first CD features a simple two-step microfluidic process and demonstrates how sequential pull-evacuation of the biosensor chamber can be implemented to replace the siphoning method normally used on microfluidic CDs. The second CD design implements an example sequence for a fluorescent immunoassay for antigen detection and describes how (i) wash, (ii) rinse, and (iii) double volume wash are successively accomplished on the platform. Lastly, a study on the effectiveness of the method using immunoassay reagents is also carried out.

## Material and Methods

### Experimental Setup

**Fig 1:**
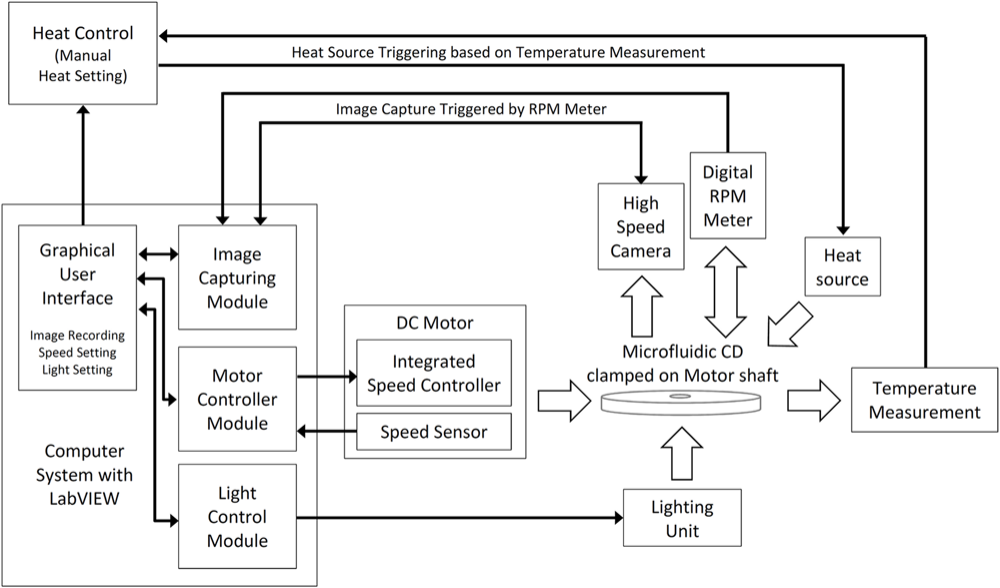
Schematic diagram of the CD spin test system. The custom made CD spin test system consists of a motorized spinning module, digital rpm meter, and high speed camera all controlled and monitored with a computer configured with LabVIEW. Forced convection heating and CD surface temperature measurement are performed using a modified industrial grade hot-air gun and infrared (IR) thermometer.

In-house fabricated microfluidic CDs were tested using a custom built CD spin test system. The CD spin test system has a motorized spinning module that is controlled by a specialized computer system equipped with LabVIEW software. Image capture is performed with an attached high-speed camera that is triggered by a digital rpm meter at a rate of one image per revolution. A schematic diagram of the CD spin test system is shown in Fig 1.

For TP pumping, a modified hot-air gun (model: Bosch GHG 630 DCE) with a built-in digital temperature controller was employed to apply forced convection heating. A focussing nozzle fitted at the end of the hot air gun provided for directed heating onto the CD surface. For CD surface temperature measurement, an Infrared (IR) thermometer (model: Smart Sensor AR550) was used. Note that a major benefit of forced convection heating compared to infra-red (IR) or laser heating is that the heat remains more localized onto the TP features close to the top CD surface, while the microfluidic layers nearer to the bottom layers are heated considerably less. The latter is important for biocompatibility of the TP pumping method [14]; convective heating tests in previous work showed that the heating of the TP air chamber (on top) and the biosensor chamber (at the bottom) are respectively 80% and 22% relative to the heating of the CD surface (e.g. while the CD surface temperature is heated from room temperature of 25°C to 50°C, the TP air chamber temperature is 45°C (80% increase), while the biosensor chamber temperature is only 30.5°C (22% increase)). This makes the process more compatible with biological / heat sensitive processes. One added advantage the forced convection method provides is that it can handle both active heating and active cooling and this can speed up several CD based assay steps considerably.

All experimental work was carried out under ambient temperatures of 25°C, and all CDs were air cooled to 25°C prior to the start of every experiment. Colored de-ionized water (prepared at a ratio of one part food dye to one hundred parts de-ionized water) was used as test liquids for the proof of concept demonstrations. Three CDs were tested to ensure the repeatability for each of the proof of concept CD designs, while five CDs were tested to determine the effectiveness of the push-wash and pull-evacuation method. Each CD contains at least 4 sets of tests according to the design dimensions. All test liquids were loaded onto the CD using pipettes following the same procedure as used in standard diagnostics labs.

### Microfluidic CD Fabrication

**Fig 2:**
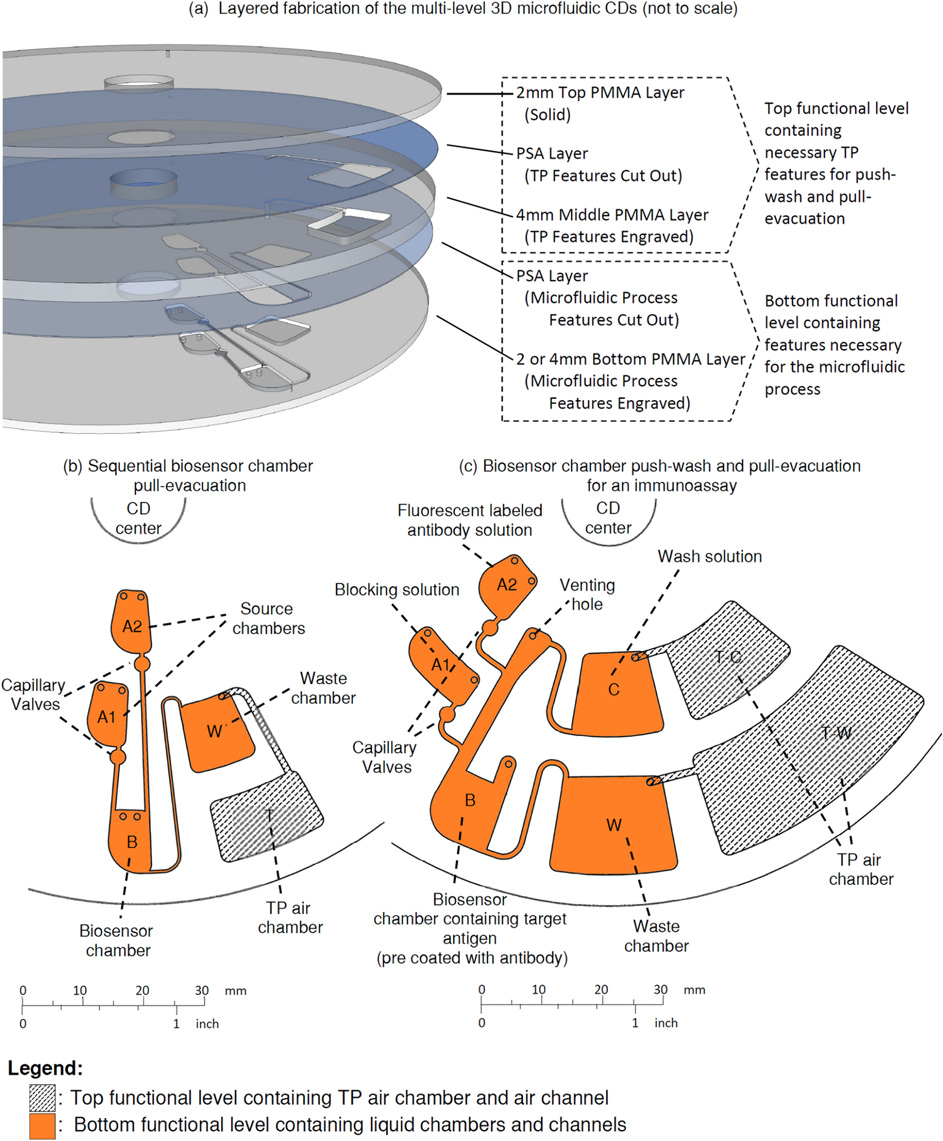
Microfluidic CD layers and demonstration CD designs. **(a)** Layered fabrication of multi-level 3D microfluidic CDs. **(b)** A design to demonstrate sequential biosensor chamber pull-evacuation. Liquid bursts from source chamber A1 into biosensor chamber B, then pull-evacuated into waste chamber W, followed by liquid bursting from source chamber A2 into biosensor chamber B, then pull-evacuated into waste chamber W. **(c)** A design to demonstrate biosensor chamber push-wash and pull-evacuation for an immunoassay. Target antigen in biosensor chamber B is washed off into waste chamber W, followed by the bursting of the blocking solution from source chamber A1 into biosensor chamber B, then rinsed and washed off into waste chamber W, and finally the bursting of flourescent labelled antibody solution from source chamber A2 to biosensor chamber B, then rinsed and double volume washed into waste chamber W

All microfluidic CDs utilized in this study were fabricated using a 3D CD design consisting of five layers that consist of two distinct functional levels [14]. A top functional level contains the necessary TP features for push-wash and pull-evacuation while the bottom functional level contains the features necessary for the assay. In Fig 2(a) we show how the entire CD consists of 5 layers with three layers made of PolyMethyl MethAcrylate (PMMA) material machined using a Computer Numerical Control (CNC) machine (model VISION 2525, by Vision Engraving and Routing Systems, USA). The three PMMA layers are then bound together using two layers of transparent Pressure Sensitive Adhesives (PSA) material (by FLEXcon, USA). The PSA layers are cut using a Cutting Plotter (model: GCC P2-60 / PUMA II, by GCC, Taiwan). Once all five layers are fabricated, the layers are pressure-bound together using a custom made press roller system. In our previous work [14], similarly fabricated microfluidic CDs were tested to a temperature of up to 80°C (on the surface). For biomedical applications such as immunoassays, the CDs are only heated up to 60°C (on the surface). We estimate each PMMA CD can contain up to 5 sets of microfluidic applications, and can be produced cost effectively at current manufacturing capability.

### Microfluidic CD Fundamentals

This section introduces the theory behind pumping on a CD in general and details how TP microfluidic CD pumping is implemented.

#### Passive and active pumping

To understand the movement of liquids in the two CD designs utilized in this work, a basic understanding of the two principal types of liquid pumping, namely passive pumping and active pumping on CDs is required.

Passive pumping is based on liquid bursting through a passive valve when the CD spin speed exceeds the burst frequency of the relevant valve. The burst frequency of a passive valve (in rpm) is given as [4]:

$$rpm=\sqrt{\frac{P_{cap}}{\rho \Delta r\bar{r}}}\left(\frac{30}{\pi }\right)$$ (1)

where *ρ* is the density of the liquid, Δ*r* is the difference between the top and bottom levels of the liquid at rest with respect to the CD center, $\bar{r}$is the average distance of the liquid from the CD center, and *P*_*cap*_ is given by [4]:

$$P_{cap}=\frac{4\cos\theta_{c}\gamma_{la}}{D_{h}}$$ (2)

where *θ*_*c*_ is the liquid contact angle, *γ*_*la*_ is the liquid-air surface energy, and *D*_*h*_ is the hydraulic diameter of the channel leading into the valve. During heating of the liquid-air interface the surface energy *γ*_*la*_ reduces as the temperature increases (eg., *γ*_*la*_ is reduced by less than 3% when the temperature is increased from room temperature to 37°C)[15]. This in turn reduces the burst frequency of a passive valve on the microfluidic CD (see Eqs 1 and 2).

In contrast, active pumping (which is employed in this work) is based on our unique TP Push Pull pumping method, where heated TP air chambers provide push pumping and cooled TP air chambers provide pull pumping. The volume that is pushed and pulled can be estimated from [14]:

$$V_{h}=k_{TP}\frac{nR(T_{h}-T_{0})+2P_{0}V_{0}-V_{0}P_{h}}{P_{0}}$$ (3)

where *V*_*h*_ is the air volume after heating, *V*_*0*_ is the initial air volume before heating, *n* is the number of moles of air that is heated, *T*_*h*_ is the temperature after heating, *T*_*0*_ is the initial temperature, *P*_*h*_ is the maximum pressure during spinning, and *P*_*0*_ is the initial pressure. As our TP pumping method does not fully obey the ideal gas law, a corrective heat factor *k*_*TP*_ needs to be determined experimentally for each design [14]. The need for the correction factor is due to a combination of design and heating / cooling factors such as (i) the ratio of heated surface to air volume in the TP air chamber that changes from one design to another, (ii) the non-uniform heating of air in the TP air chambers during pumping due to the change in air temperature as the expanding heated air expands from the heated TP air chamber into a cooler chamber on the CD, and (iii) the undesired partial heating of the air in unvented areas other than the TP air chamber (such as liquid chambers and the interconnecting channels).

#### Push-wash and pull-evacuation

**Fig 3:**
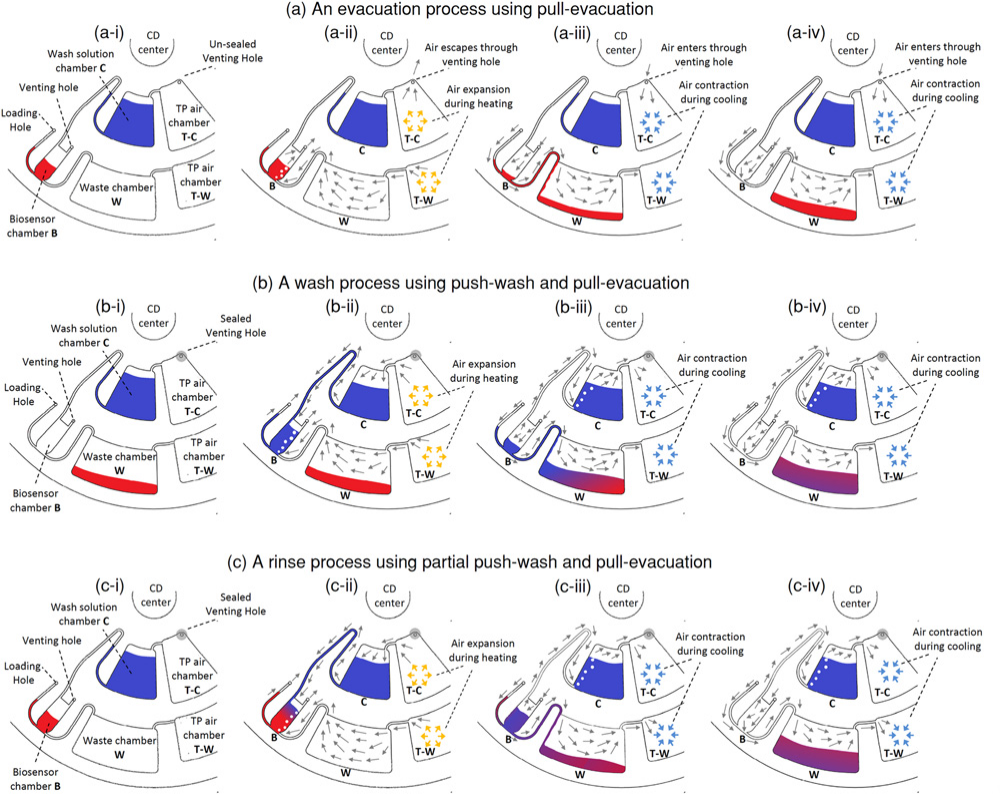
Evacuation, wash, and rinse using push-wash and pull-evacuation. **(a)** Sequence of steps for the evacuation of a biosensor chamber using pull-evacuation. **(b)** Sequence of steps for the washing of an empty biosensor chamber using a push-wash followed by a pull-evacuation. **(c)** Sequence of steps for rinsing a non-empty biosensor chamber with a partial push-wash following by a pull-evacuation.

A TP air chamber connected to a liquid chamber allows for push pumping of liquid out of a liquid chamber. Push-wash takes place when the TP air chamber is being heated thus expanding the air and pushing washing liquid out from the liquid chamber into an adjoining chamber. A liquid chamber, an empty waste chamber, and a TP air chamber that are interconnected in series allows also for pull pumping of liquid from the liquid chamber into the empty waste chamber. Pull-evacuation takes place when a preheated TP air chamber is cooled down, and contracting air then pulls the liquid from the liquid chamber into the adjoining waste chamber, thus evacuating the liquid chamber. This washing and evacuation process can be performed over and over. In Fig 3 we illustrate the sequence of steps for an evacuation, a wash, and a rinse. The CD design in Fig 3 consists of a biosensor chamber, wash solution chamber, waste chamber, and two TP air chambers. Each process starts with a blue liquid in the wash solution chamber C, and a red liquid in biosensor chamber B. The TP air chamber (T-C) contains a venting hole while the TP air chamber (T-W) is sealed. Heat is assumed to be applied uniformly over both TP air chambers when actuated.

Fig 3(a) shows the evacuation process using only pull-evacuation. To prepare for pull-evacuation, the TP air chamber T-W needs to be preheated. To ensure that the push-wash does not become actuated during this preheating stage, the venting hole above the TP air chamber T-C is left un-sealed (see Fig 3(a-i)). As the air chambers are heated, expanding air in the TP air chamber T-C is able to escape through the unsealed venting hole, while expanding air in the TP air chamber T-W expands through the waste chamber W, and then through the red liquid in the biosensor chamber B and finally escapes through the venting hole in that chamber (see Fig 3(a-ii)). Once the TP air chamber T-W is heated up, the heat is cut off and the cooling process actuates the pull-evacuation. Contracting air in the TP air chamber T-W pulls the red liquid into the biosensor chamber B through the connecting channel into waste chamber W. Meanwhile contracting air in the TP air chamber T-C simply pulls air in through the venting hole (see Fig 3(a-iii)). This process continues until all the red liquid is pulled into waste chamber W (see Fig 3(a-iv)).

Fig 3(b) demonstrates how a wash is accomplished using a pull-wash followed by a pull-evacuation. The illustrations shown are a continuation from Fig 3(a) after the evacuation process. In this process, the venting hole above the TP air chamber T-C is sealed such that both TP air chambers can be activated at the same time (see Fig 3(b-i)). As heat is applied, both TP air chambers are heated up, and the air in both chambers start to expand. The expanding air in TP air chamber T-W now pushes the blue liquid out from wash solution chamber C into biosensor chamber B (see Fig 3(b-ii)). At the same time, expanding air in TP air chamber T-W expands through the waste chamber W, and then through the red liquid in biosensor chamber B and escapes through the venting hole. Once sufficient blue liquid fills biosensor chamber B, the heat is cut off and the cooling process then activates pull-evacuation. The blue liquid from biosensor chamber B is then pulled into waste chamber W (see Fig 3(b-iii) and 3(b-iv)).

To perform a wash immediately after an evacuation requires stopping the CD to seal the venting hole of the TP air chamber T-C. This is not desirable as it disrupts the automation of the microfluidic process. To avoid stopping the CD, the CD may have both TP air chambers sealed from the start, and the initial heating of the CD then actuates the push-wash while preparing for the pull-evacuation. In situations where there is still liquid in the biosensor chamber (not yet evacuated), this results in a rinse process. In Fig 3(c) we describe a rinse process where both TP-air chambers are actuated while there is still red liquid in the biosensor chamber B (see Fig 3(c-i) and 3(c-ii)). This results in some blue liquid topping up onto the red liquid in biosensor chamber B. Once the heat is cut off, and the cooling process starts, the mixture of blue and red liquid will be pulled into the waste chamber W (see Fig 3(c-iii) and 3(c-iv)). The rinse process is basically a partial wash followed by a full evacuation.

### CD Designs

To demonstrate how push-wash and pull-evacuation can be implemented for biosensor chambers on microfluidic CDs, two CDs were designed and fabricated (see Fig 2(b) and 2(c)). The first CD design demonstrates how sequential pull-evacuation of a biosensor chamber can be accomplished with ease. As shown in Fig 2(b), the top functional level of the CD contains the TP features (TP air chamber and connecting channel), while the bottom level contains the microfluidic features (source chambers A1 and A2, biosensor chamber B, and waste chamber W). TP air chamber T (with a 160 mm^3^ volume capacity) is connected to waste chamber W. Source chambers A1 and A2 are designed to have different burst frequencies. In this demonstration, liquids from source chambers A1 and A2 are subsequently burst into biosensor chamber B, and sequentially evacuated into waste chamber W through repeated heating and cooling of the TP air chamber T. Details of how pull-evacuation is performed are given in Section 3. Results and Discussion.

The second microfluidic CD design constitutes an example sequence for an immunoassay for antigen detection (see Fig 3(b)). The top functional level of the CD contains the TP features (TP air chambers T-C and T-W and connecting channels), while the bottom level contains the necessary features to perform an immunoassay (source chambers A1 and A2, biosensor chamber B, wash solution chamber C, and waste chamber W). Source chambers A1 and A2 are designed to have different burst frequencies. TP air chamber T-C (with a 200 mm^3^ volume capacity) is connected to wash solution chamber C. To perform push-wash, TP air chamber T-C is heated up to push wash solution out of wash solution chamber C into the connected biosensor chamber B. TP air chamber T-W (with a 600 mm^3^ volume capacity) is connected to waste chamber W. To perform pull-evacuation, TP air chamber T-W is preheated and then cooled down to pull any liquid in biosensor chamber B into waste chamber W. To illustrate how an actual immunoassay such as a fluorescent immunoassay can be performed, various colored liquids are used to represent samples containing a target antigen, blocking solution, and fluorescent labelled antibody, and the biosensor chamber is assumed to be pre-coated with capture antibodies or contain a biosensor chip. Details on how push-wash and pull-evacuation is performed in an immunoassay, and how (i) wash, (ii) rinse, and (iii) double volume wash of the biosensor chamber is accomplished, as well as the effectiveness of the wash are discussed in detail in Section 3. Results and Discussion.

The microfluidic CDs were designed taking into consideration fabrication errors and tolerances, as well as contamination risks. Fabrication error and tolerances can lead to various issues such as venting hole blockage or passive valve failure. Although in an automated fabrication setup these issue are often not encountered at all, it can be an issue in a manual prototyping setting with poor workmanship. In our current and previous work we have had good success in producing CDs with burst frequency tolerances of ±10%. Designing all successive burst steps to be further than 20% from each other mitigates the issue of unintentional mixing of liquids due to premature bursting of passive valves. Another consideration for a passive valve is the minimum dimension for effective valving. Experimentally, we have determined that the capillary valve must have a minimum diameter equivalent to 3 times that of the width of the channel it is placed over (see Fig 2(b) and 2(c)).

To minimize contamination due to overflowing of liquid from the biosensor chamber, the chamber can be designed to handle two successive bursts of liquid (i.e., the chamber is large enough to accommodate a combined liquid from two bursts). This can be done by increasing the chamber size, or also by extending the channel leading to the venting hole further away from the chamber. Another consideration for minimizing contamination is the size and positioning of the venting and loading holes. The holes should be 1mm or less in diameter to ensure sample introduction using a pipette can be performed while the liquid will not flow out of it due to the interfacial energy of the hole. Also, to avoid spillage, venting holes must be positioned at least 1mm above the liquid level when a chamber is full.

## Results and Discussion

The results and discussion section is separated into three sub-sections. The first two sections describe the demonstration of the two CD designs shown in Fig 3(b) and 3(c), and the third section discusses the effectiveness of the push-wash and pull-evacuation in performing chamber washing for biological applications compared to conventional bench top pipetting washing method.

### Sequential Biosensor Chamber Pull-Evacuation

**Fig 4:**
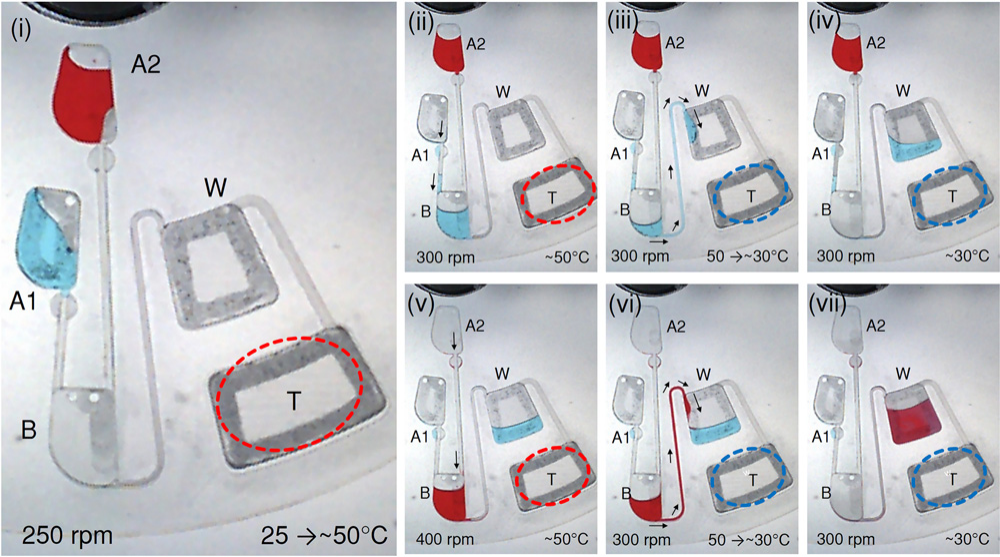
Demonstration of sequential biosensor chamber pull-evacuation: **(i)** Blue and Red liquids are loaded into source chamber A1 and A2. **(ii—iv)** Blue liquid bursts from source chamber A1 into biosensor chamber B, then pull-evacuated into waste chamber W. **(v—viii)** Sequentially Red liquid bursts from source chamber A2 into biosensor chamber B, then pull-evacuated into waste chamber W

The demonstration of sequential biosensor chamber pull-evacuation is shown in Fig 4. The process demonstrates how two liquids that burst at separate times into biosensor chamber B can be sequentially pull-evacuated into waste chamber W. Fig 4(i) illustrates the initiation of the test with the loading of source chambers A1 & A2 with 40μL of Blue and Red colored liquids respectively. Next, the microfluidic CD is spun up gradually to 250 rpm, and the heat source is positioned over the TP air chamber T and powered ON to prepare it for pull-evacuation. During the heating process, the heated air in TP air chamber T expands and escapes through the venting holes in biosensor chamber B. Once the CD surface reaches 50°C (after about 4 minutes), the CD spin speed is gradually increased to 300rpm to burst the Blue liquid from source chamber A1 into biosensor chamber B (see Fig 4(ii)). The heat source is then powered OFF, and the CD is allowed to cool down while rotating at 300rpm. Fig 4(iii) represents the stage at which the CD starts to cool down and the trapped air in TP air chamber T starts to contract and pulls the Blue liquid from biosensor chamber B towards waste chamber W. In Fig 4(iv), the evacuation of the Blue liquid is completed in approximately 4 minutes.

To repeat this process with the Red liquid, the CD is once again heated to 50°C, then the CD spin speed is gradually increased to 400rpm to burst the Red liquid from source chamber A2 into biosensor chamber B (see Fig 4(v)). Once the heat source is powered OFF, the Red liquid is then pull-evacuated from biosensor chamber B into waste chamber W as shown in Fig 4(v) and 4(vi). This second heating of the TP air chamber T takes around 2 minutes, and the transfer of the Red liquid to waste chamber W takes approximately 4 minutes, making the total time required for the entire process to be about 14 minutes.

Sequential biosensor chamber pull-evacuation presents an alternative to the siphoning method normally used in transferring liquids from biosensor chambers to waste chambers. Whereas waste chambers traditionally occupy the outer most space on a CD, sequential pull-evacuation allows for the waste chamber to be closer to the CD center relative to the biosensor chamber. Furthermore, in a multi-level 3D CD, the waste chamber can even be placed in an overlapping position with the original microfluidic process, allowing for the valuable space near the CD edge to be used for other steps within a microfluidic process.

### Push-wash and Pull-evacuation in an Immunoassay

**Fig 5:**
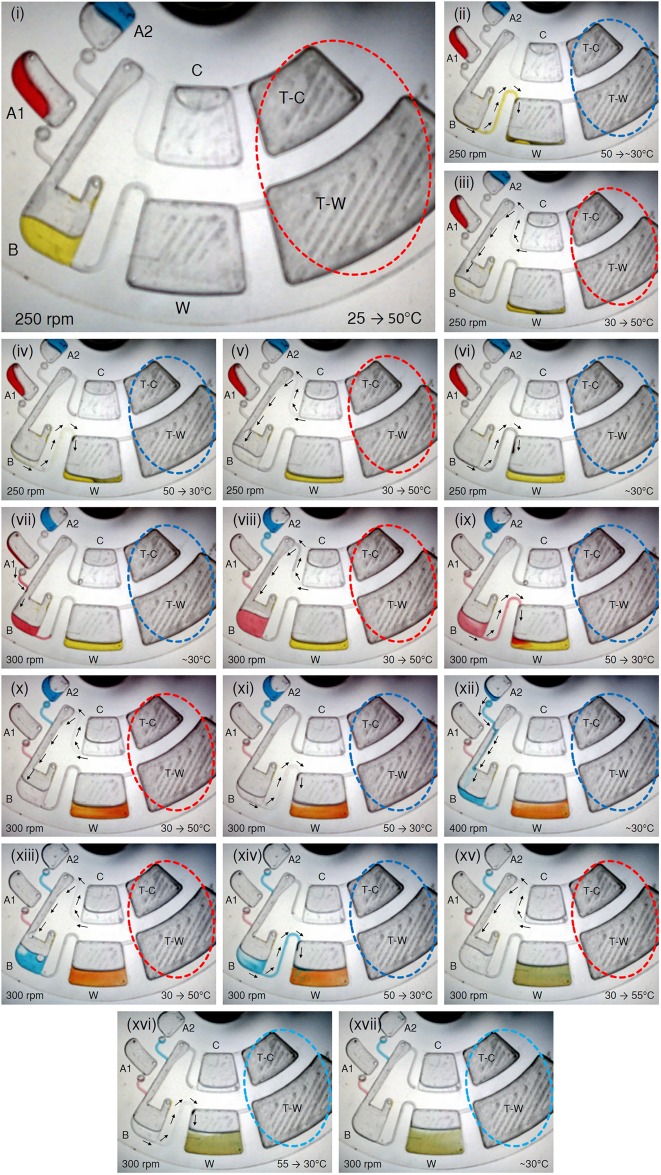
Demonstration of biosensor chamber push-wash and pull-evacuation for an immunoassay. **(i)** Yellow (representing test sample containing target antigen), Red (representing blocking solution), Blue (representing fluorescent labelled secondary antibodies) liquids, and de-ionized water (representing washing solution) are respectively loaded into biosensor chamber B, source chambers A1 and A2, washing solution chamber C. **(ii—vi)** Yellow liquid is pull-evacuated into waste chamber W, wash solution chamber is sealed and biosensor chamber is washed twice. **(vii—xi)** Red liquid is burst into biosensor chamber B, followed by a rinse and a wash of the biosensor chamber B. **(xii—xvii)** Blue liquid is burst into biosensor chamber B, followed by a rinse and a double volume wash of the biosensor chamber B.

The demonstration of how biosensor chamber push-wash and pull-evacuation for an antigen detection immunoassay can be implemented on the microfluidic CD is shown in Fig 5. Fig 5(i) illustrates the initiation of the test with the loading of biosensor chamber B and source chambers A1 & A2 with 60μL of Yellow, Red and Blue colored liquids respectively, and the washing solution chamber C with 420 μL of de-ionized water. To relate this to an actual antigen detection fluorescent immunoassay, imagine that the CD is made of black PMMA material, biosensor chamber B is pre-coated with capture antibodies, Yellow liquid represents test samples containing the target antigen, Red liquid represents the blocking solution, and Blue liquid represents the fluorescently labelled secondary antibodies. The sequence of the immunoassay is then as follows: the test sample containing the target antigen is pull-evacuated into waste chamber W, and biosensor chamber B is then push-washed twice; next the blocking solution is burst into biosensor chamber B and is then pull-evacuated into waste chamber W followed by push-washes; finally the fluorescent labelled antibody solution is burst into biosensor chamber B and subsequently pull-evacuated into waste chamber W, and then the biosensor chamber is push-washed twice. Note that for simplicity in the discussion, incubation steps are not included, but should be included in an actual immunoassay [16].

As the proposed design demonstrates three microfluidic processes for the biosensor chamber: wash, rinse, and double volume wash (each constituting a push-wash followed by a pull-evacuation), the discussion here is separated into three parts. *Part I* explains the evacuation of Yellow liquid (test sample) and the washings of biosensor chamber B, *Part II* describes the bursting of Red liquid (blocking solution) into biosensor chamber B and the rinse and wash of biosensor chamber B, and *Part III* discusses the bursting of Blue liquid (fluorescently labelled secondary antibody) into biosensor chamber B and the rinse and double volume wash of biosensor chamber B.

*Part I* starts with the spinning of the CD up to 250 rpm while being heated to 50°C to prepare for pull-evacuation of the Yellow liquid (test sample) (see Fig 5(i)). This process takes approximately 2 minutes and then the heat source is powered OFF. Note that in this test, both TP air chambers are heated and cooled at the same time. When both TP air chambers are heated, the heated air in TP air chamber T-C expands through the connected channel to push wash solution out of chamber C into biosensor chamber B; on the other hand, the heated air in TP air chamber T-W simply escapes through the venting holes in biosensor chamber B. Subsequently cooling both TP air chambers causes the trapped air in TP air chamber T-W to contract and pull any liquid in biosensor chamber B into waster chamber W; in contrast, the shrinking air in TP air chamber T-C pulls air from venting holes at the top of biosensor chamber B into TP air chamber T-C (through the channel connecting chamber B and chamber C, then through the wash solution in chamber C). Initially the wash solution chamber C is left unsealed (by leaving the loading hole unblocked) to ensure that push-wash does not happen while the TP air chamber T-W is preheated for pull-evacuation of the Yellow liquid. As shown in Fig 5(ii), once the heat source is powered OFF, the CD is allowed to cool down to 30°C and the Yellow liquid is pull-evacuated from the biosensor chamber into waste chamber W. Once pull-evacuation is completed, the CD is temporary stopped and the loading hole of wash solution chamber C is sealed to prepare for the wash process. The heat source is then powered ON and wash solution is pushed from chamber C into biosensor chamber B as shown in Fig 5(iii). This process continues for about 1 minute and the CD surface temperature is measured to be 50°C at the end of the process. Once the push-wash is completed, the heat source is powered OFF and the CD is left to cool down to 30°C to allow the pull-evacuation of the wash solution from biosensor chamber B to waste chamber W to take place (see Fig 5(iv)). This step takes approximately 2 minutes. Once the first wash is completed, a second push-wash and pull-evacuation is performed by repeating the heating and cooling of the TP air chambers as shown in Fig 5(v) and 5(vi). In this test, each subsequent wash cycle took around 3 minutes (1 minute for push-wash, and 2 minutes for pull-evacuation), and the heating and cooling cycles were bound between temperatures of 30°C and 50°C. Note that even though the TP air chamber T-W used for pull-evacuation is bigger than the TP air chamber T-C used for push-washing, the pull-evacuation process takes longer because push-wash takes place nearer to the CD center and experiences less centrifugal force against liquid flow than pull-evacuation which is nearer to the CD edge which experiences more centrifugal force.

*Part II* starts with the bursting of the Red liquid from source chamber A1 into biosensor chamber B by increasing the rpm to 300 as shown in Fig 5(vii). Next the heat source is powered ON and the CD is heated to 50°C to actuate the pushing of wash solution from chamber C into biosensor chamber B. Unlike the washing in *Part I*, after the wash solution chamber C is sealed, any preheating to prepare for a pull-evacuation step triggers a push-wash. As shown in Fig 5(viii) this push-wash is actually a rinse that effectively dilutes the Red liquid prior to the pull-evacuation step. This rinsing process continues for approximately 1 minute before the heat source is powered OFF. During cooling the diluted Red liquid is pull-evacuated into waste chamber W (see Fig 5(ix)), which takes approximately 2 minutes. Once this is done, a proper wash is performed by repeating the heating and cooling cycle as shown in Fig 5(x) and 5(xi).

At the start of *Part III*, the CD is spun to 400 rpm to burst the Blue liquid from chamber A2 into biosensor chamber B (see Fig 5(xii)). Immediately after bursting of the Blue liquid into the biosensor chamber the CD is spin down back to 300 rpm and the heat source is powered ON to prepare for the rinsing process. Fig 5(xiii) shows the rinsing process in which the Blue liquid gets diluted. An interesting observation here is that bubbles due to air escaping from the T-W via waste chamber W stir up the diluting liquid and this allows for better cleaning of the biosensor chamber. Once the rinse process is completed, the heat source is powered OFF and the Blue liquid is pull-evacuated from biosensor chamber B into waste chamber W (see Fig 5(xiv)). To demonstrate that the wash volume can easily be controlled, a double volume wash was performed as shown in Fig 5(xv). A double volume push-wash was achieved by powering the heat source ON for 2 minutes, and subsequently, powering OFF the heat source to start the pull-evacuation process, which completes within 2 minutes. Note that even though the volume has doubled the pull-evacuation process takes the same time as before because the higher temperature range during the cooling process results in greater air contraction in TP air chamber T-W. Once the heat source is powered OFF the Blue liquid is pull-evacuated from biosensor chamber B into waste chamber W (see Fig 5(xvi) and 5(xvii)). The entire process described above takes approximately 20 minutes.

### Wash Performance Evaluation

To implement automated push-wash and pull-evacuation as a viable washing method for actual immunoassays, the performance needs to be comparable to conventional manual washing methods. To test this, a set of preliminary experiments was conducted using the CD design shown in Fig 3. The biosensor chamber was first loaded with a 60μl solution of mouse monoclonal anti-dengue immunoglobulin type M antibodies (IgM) conjugated to horseradish peroxidase (HRP). To simulate an actual immunoassay process, the CD was incubated at 37°C for 60 minutes. Next the biosensor chamber was subjected to several combinations of evacuation, rinse, and wash carried out using the push-wash and pull-evacuation method as described in **Section 2.3.2** to wash away the anti-Dengue IgM HRP conjugate solution. Each wash was performed with 120μl of PBS washing solution, while each rinse was performed with approximately 60μl of PBS washing solution (as each rinse was performed when the biosensor chamber still contained 60μl of anti-Dengue IgM HRP conjugate solution). Once this was completed, 100μl of Hydrogen peroxidase Tetramethylbenzidine (TMB) solution was pipetted into the biosensor chamber. The CD was covered to prevent exposure to light since TMB degrades upon exposure and then left at room temperature for 10 minutes. Finally 100μl of 1.6N sulfuric acid as stopping solution was added into the biosensor chamber and the CD was left again at room temperature for 10 minutes. The mixture in the biosensor chamber was then pipetted out and transferred into a 96 microwell plate and measured at 450nm in a plate reader. Note that in the last two steps the TMB and stopping solution were both 100μl because the plate reader is optimized for reading solution volumes of 200μl in the microwell plate.

Any unwashed HRP left in the biosensor chamber will react with the TMB solution, giving it a blue hue, and once the stopping solution is added, the blue hue changes to yellow. For comparison purposes two other tests were conducted as follows: (i) a manual test in which the entire experiment is carried out in microwells using manual pipette evacuation and washing, and (ii) a test where the biosensor chamber is empty and only the TMB solution and the stopping solution are added at the end. While the first test replicates the manual washing performed in actual immunoassays, the second test represents an ideal case of perfect washing, and is only for reference purposes.

**Fig 6:**
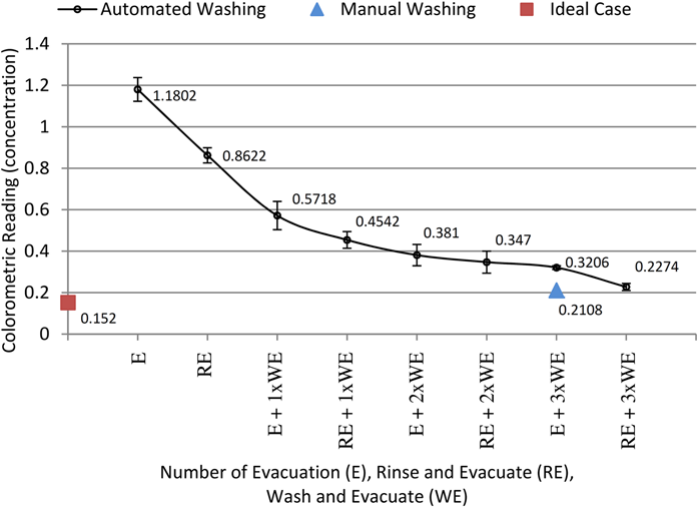
Performance evaluation of push-wash and pull-evacuation. Performance evaluation of implementing evacuation, rinse and wash using push-wash and pull-evacuation in an immunoassay.

The performance of various combinations of evacuation (E—consisting of a pull-evacuation), rinse (RE—consisting of a partial push-wash and pull-evacuation) and wash (WE—consisting of a push-wash and pull-evacuation) are shown in Fig 6. A low concentration of 0.152 (absorbance value) marks an ideal perfect wash while a high concentration of 1.1802 indicates no washing. As shown, using only an evacuation and no rinse or wash (see E in Fig 6), the concentration is the highest at 1.1802 while an automated wash (RE + 3xWE in Fig 6), and the manual wash (E + 3xWE in Fig 6) produce two of the lowest relative concentrations values of 0.2274 and 0.2108 respectively. As the biosensor chamber is repeatedly push-washed and pull-evacuated, the concentration progressively drops closer to the ideal value, indicating a more effective wash. Comparing experiments that start with an evacuation (E) with those that start with a rinse and evacuation (RE), a rinse and evacuation provides better overall washing. It can be seen that a rinse and evacuation, followed by three consecutive washes and evacuations (RE + 3xWE in Fig 6) produces a concentration value that is close to the manual wash (E + 3xWE in Fig 6). This result is encouraging as it is easier to perform a rinse and evacuation (RE) first, followed by numerous wash and evacuation (WE) than to first perform an evacuation (E) followed by numerous wash and evacuation (WE) (as explained in section 2.3,2, it is necessary to stop the CD to seal the TP air chamber when switching over from an initial evacuation (E) to a wash and evacuate (WE)).

In general, both the manual wash and the automated wash (RE + 3xWE in Fig 6) are not able to obtain the same result as the ideal case. The possible reason could be either some residue left in the chamber / microwell in either case, dead volume in the chamber, or surface absorption of the HRP marker. However, as immunoassay results are analyzed based on a threshold method (where a test is considered positive if the final concentration of the test exceeds a calculated mean value using negative controls), the washing method does not need to produce ideal perfect results. As the result for the automated wash (RE + 3xWE in Fig 6) is within 10% of the manual wash (which replicates bench top washing performed in actual immunoassays), the automated wash can be successfully implemented in actual immunoassays to replace the tedious manual washing method.

## Conclusions

We have presented a push-pull pumping mechanism for sequential push-wash and pull-evacuation of a biosensor chamber on a microfluidic CD platform. Employing a pair of adjacent TP air chambers, one for push-wash and one for pull-evacuation, numerous rinse or washes (each consisting of a push-wash followed by a pull-evacuation) could be simply initiated by consecutive heating and cooling cycles.

Two applications of the push-wash and pull-evacuation were demonstrated. The first demonstration of sequential pull-evacuation showed how pull-evacuation can be applied to replace the siphoning method normally used in transferring liquids from biosensor chambers to waste chambers on microfluidic CDs. The second demonstration was of an example sequence for an immunoassay with wash, rinse and double volume wash that were implemented using push-wash and pull-evacuation.

These demonstrations show that push-wash and pull-evacuation provide a superior alternative to conventional washing on a microfluidic CD platform that requires repetitive fillings of wash solution from multiple chambers (which is limited by the number of passive valves which can be designed on a space constraint CD) and emptying of the biosensor chamber via siphoning (which is rotational speed dependent, requires a hydrophilic channel to operate, and has repeatability issues). In addition, by implementing a multi-level 3D CD, disc space usage was optimized for push-wash and pull-evacuation, and efficient heating was performed with the TP air chambers on the top level while the heat sensitive biosensor chamber were on the lower level. A further performance evaluation showed that a biosensor chamber implemented using a rinse and evacuate, followed by three washes and evacuations (RE + 3xWE) was comparable to that of a manual wash performed using a bench top pipetting technique. This demonstrates that the method is suitable for implementation in actual immunoassays.

The demonstrations and evaluation tests showed that the push-wash and pull-evacuation techniques are easy to implement, can be actuated on demand, are reliable, and can easily be adapted to various multi-stepped processes by simply modifying the number of source chambers and the volume of the wash solution chamber. Furthermore the technique introduces a heat source that is readily available for incubation processes, and still ensures the portability of the CD platform with no additional physical connections needed. The washing method described in this work will be implemented onto a multi-stepped microfluidic CD towards the development of a portable diagnostic device.
